# Water Level Has Higher Influence on Soil Organic Carbon and Microbial Community in Poyang Lake Wetland Than Vegetation Type

**DOI:** 10.3390/microorganisms10010131

**Published:** 2022-01-09

**Authors:** Qiong Ren, Jihong Yuan, Jinping Wang, Xin Liu, Shilin Ma, Liyin Zhou, Lujun Miao, Jinchi Zhang

**Affiliations:** 1 Co-Innovation Center for Sustainable Forestry in Southern China, Jiangsu Province Key Laboratory of Soil and Water Conservation and Ecological Restoration, Nanjing Forestry University, Nanjing 210037, China; jane5872@126.com (Q.R.); liuxinswc@gmail.com (X.L.); supermsl@163.com (S.M.); 2 Wetland Ecological Resources Research Center, Jiangxi Academy of Forestry, Nanchang 330032, China; yuanjh040@nenu.edu.cn (J.Y.); zly201021@126.com (L.Z.); miaolujun@126.com (L.M.); 3 Jiangxi Key Laboratory for Restoration of Degraded Ecosystems and Watershed Ecohydrology, Nanchang Institute of Technology, Nanchang 330099, China; wangjp0107@nit.edu.cn

**Keywords:** wetland soil, organic carbon characteristics, microbial diversity and abundance, water level and vegetation type

## Abstract

Although microorganisms play a key role in the carbon cycle of the Poyang Lake wetland, the relationship between soil microbial community structure and organic carbon characteristics is unknown. Herein, high-throughput sequencing technology was used to explore the effects of water level (low and high levels above the water table) and vegetation types (*Persicaria hydropiper* and *Triarrhena lutarioriparia*) on microbial community characteristics in the Poyang Lake wetland, and the relationships between soil microbial and organic carbon characteristics were revealed. The results showed that water level had a significant effect on organic carbon characteristics, and that soil total nitrogen, organic carbon, recombinant organic carbon, particle organic carbon, and microbial biomass carbon were higher at low levels above the water table. A positive correlation was noted between soil water content and organic carbon characteristics. Water level and vegetation type significantly affected soil bacterial and fungal diversity, with water level exerting a higher effect than vegetation type. The impacts of water level and vegetation type were higher on fungi than on bacteria. The bacterial diversity and evenness were significantly higher at high levels above the water table, whereas an opposite trend was noted among fungi. The bacterial and fungal richness in *T. lutarioriparia* community soil was higher than that in *P. hydropiper* community soil. Although both water level and vegetation type had significant effects on bacterial and fungal community structures, the water level had a higher impact than vegetation type. The bacterial and fungal community changes were the opposite at different water levels but remained the same in different vegetation soils. The organic carbon characteristics of wetland soil were negatively correlated with bacterial diversity but positively correlated with fungal diversity. Soil water content, soluble organic carbon, C/N, and microbial biomass carbon were the key soil factors affecting the wetland microbial community. Acidobacteria, Alphaproteobacteria, Verrucomicrobia, Gammaproteobacteria, and Eurotiomycetes were the key microbiota affecting the soil carbon cycle in the Poyang Lake wetland. Thus, water and carbon sources were the limiting factors for bacteria and fungi in wetlands with low soil water content (30%). Hence, the results provided a theoretical basis for understanding the microbial-driven mechanism of the wetland carbon cycle.

## 1. Introduction

Wetlands comprise swamps, bogs, peatlands, and water areas (including coastal areas with a water depth of less than 6 m at low tide), accounting for 2–6% of the total land area of the world, and stores one-third of the global soil organic carbon (SOC). They play an irreplaceable role in regulating water sources, controlling runoff, purifying water quality, fixing carbon and releasing oxygen, improving climate, cycling nutrients, maintaining regional ecological balance, and providing biological habitat [1]. In addition, wetlands play an important role in the global carbon cycle. Small changes in wetland soil carbon pool can lead to significant alterations in atmospheric CO_2_ content, ultimately resulting in climate change [1,2]. At present, with global warming, increasing attention has been paid to the problem of carbon emission. As an important part of global carbon storage, the wetland has become an essential carbon sink owing to its low decomposition rate of organic matter and high productivity [2]. However, under the influence of climate change and human activities, wetlands can change from carbon sink to carbon source and release greenhouse gases to the atmosphere [3]. Therefore, the study of wetland soil carbon has become crucial. Research on wetland soil carbon mainly focuses on the distribution and variation of SOC, carbon source/sink balance, components of organic carbon, carbon cycle, and factors influencing organic carbon components [3,4]. The core topic of these works is the input and output of organic carbon. When the input of organic carbon was higher than the output, accumulation of wetland organic carbon occurred, which has immense significance in alleviating global warming.

The main factors affecting the accumulation of organic carbon in wetlands are climate, hydrology, soil, and vegetation [5]. Among them, soil, which is one of the three elements and core of the wetland ecosystem, is the main site for storing organic carbon. As an important part of soil, soil microorganisms have a key function in soil nutrient cycling, carbon and nitrogen fixation, organic matter decomposition, and material and energy cycle [6]. Therefore, soil microorganisms have an irreplaceable role in maintaining wetland ecological balance, and their community structure and diversity have a profound impact on wetland biodiversity and functional stability. Furthermore, soil microorganisms are the drivers of the key process of the soil carbon cycle, which play an important role in wetland carbon fixation [4]. Microbial residues are an important source of SOC [7], and autotrophic carbon-fixing bacteria are the main driving factor of carbon fixation in wetlands [8]. Furthermore, microbial activities affect the decomposition and mineralization of SOC [9].

The effect of microorganisms on wetland carbon fixation is controlled by many factors, with groundwater level and vegetation type being the major factors on a regional scale. The impact of water level fluctuation on soil microorganisms is the key to determining the carbon cycle of the wetland ecosystem [10]. The change in water level can affect the distribution of soil microorganisms in wetlands, alter the microbial community, regulate microbial activities, and finally affect the decomposition of organic matter. However, the microbial-driven mechanism of the effect of water level change on soil carbon regulation is not clear, and relevant research results are not consistent. Previous studies have shown that the increase in the water level could inhibit plant root respiration, reduce soil microbial activity, and inhibit microbial decomposition of organic carbon, which is conducive to carbon fixation [10,11]. However, some studies have demonstrated that the carbon content of soil microbial biomass increased after the increase in water level, and long-term flooding enhanced microbial activity and promoted the cyclic transformation of microorganisms to organic matter [12,13]. Therefore, the effect of water level change on microorganisms must be further investigated. Vegetation can affect soil physical-chemical properties as well as soil microorganisms by providing oxygen, litter, and root exudates, ultimately influencing SOC accumulation in wetlands [14]. Owing to the variation in litter quantity, decomposition rate, and relative content of root exudates, different vegetation types lead to differences in the structure and function of the soil microbial community [15,16]. Although water level and vegetation type have important influences on wetland soil microorganisms and organic carbon, current research does not pay much attention to comparing these two factors. Owing to their immense diversity, different kinds of microorganisms exhibit varied responses to water level and vegetation changes. Therefore, determining the microbial community changes at different water levels and different vegetation types could help to understand the microbial-driven mechanism of the wetland soil carbon cycle.

The Poyang Lake wetland is a large-area dry-wet alternating beach wetland formed by the combined action of water from the five major basins and water level of the Yangtze River [17]. As an internationally important wetland, it plays an important role in global carbon storage [18]. Since the middle of the 20th century, Poyang Lake wetland has been affected by climate change and human activities (such as water conservancy projects, trenching and drainage, reclaiming farmland from the lake, overgrazing, etc.), which have resulted in a series of ecological and environmental problems, including a significant reduction in wetland area, advancement and extension of the exposure time of wetland beach, soil degradation, change in the plant community, and deterioration of water quality. As a result, the ecological balance of the Poyang Lake wetland has been seriously disturbed [17]. A significant reduction in wetland area can lead to a substantial decrease in the carbon storage function of Poyang Lake and exacerbate climate warming. Microorganisms are sensitive to environmental changes and play an important role in soil carbon fixation in wetlands. Previous studies have revealed that the soil microbial community in the Poyang Lake wetland changed with variations in the water level [19] and vegetation [20]. However, to date, no comparative research on the effects of water level and vegetation type on soil microbial community in Poyang Lake wetland has been conducted. Moreover, the key microbial communities closely related to SOC characteristics have not yet been determined. The environmental gradient zone close to the water body of Poyang Lake wetland has been reported to present a fast turnover rate of soil carbon and nitrogen and is a key area of the wetland biogeochemical cycle [21]. Therefore, in this study, areas close to the water body of the Poyang Lake wetland were selected as the research sites. High-throughput sequencing technology was applied to investigate the changes in soil microbial community structure and diversity as well as soil carbon characteristics in response to different vegetation types at diverse soil water levels. The objectives of this study were to: (1) reveal the response of SOC characteristics and microbial community structure to the changes in water level and typical vegetation type in the Poyang Lake wetland; (2) determine the key soil factors affecting the structure and diversity of wetland soil microbial communities; and (3) identify the major microorganisms closely related to the organic carbon characteristics of wetland soil.

## 2. Materials and Methods

### 2.1. Experimental Site

**Figure 1:**
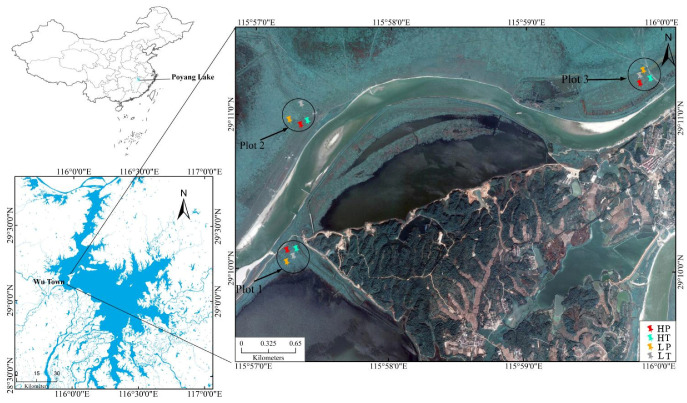
Soil sampling sites in Poyang Lake wetland. HP denotes *P. hydropiper* community at high level above the water table, HT indicates *T. lutarioriparia* community at high level above the water table, LP signifies *P. hydropiper* community at low level above the water table, and LT denotes *T. lutarioriparia* community at low level above the water table.

The Poyang Lake Basin, located in the north of Jiangxi Province, has a subtropical warm and humid monsoon climate. It is a composite multi-type wetland system composed of lakes, deltas, rivers, alluvial plains, and swamps [22]. The study area is located in Wutown (29°05′–29°15′ N, 115°55′–116°03′ E) in the Poyang Lake Basin, which is a delta front formed by the alluvial deposition of the open water in Poyang Lake from Ganjiang River and Xiuhe River (Figure 1). This region forms a peninsula surrounded by water on three sides and is composed of a large area of lakes and grassland. It presents the characteristics of a typical natural freshwater lake wetland ecosystem, with an area of about 31 km^2^, annual average temperature of 17.2 °C, and annual average precipitation of 1570 mm [23]. Affected by the seasonal hydrological changes in Poyang Lake, all the rivers, lakes, and grassland in the island are flooded in the wet season, which is a typical lacustrine hydrological phase. During the dry season in October, the lake drops to the river channel and some dished depressions. The area comprises many rivers and lakes, and beaches with different elevations are exposed one after another. The entire delta presents a natural wetland landscape of rivers, lakes, and continents. The typical vegetation types of the wetland include *Triarrhena lutarioriparia*, *Persicaria hydropiper*, *Phragmites communiss*, *Carexcinerasce,* etc., which are usually distributed in blocks or strips. 

### 2.2. Experimental Design and Sampling

Three sample plots along both sides of the river were established. In each sample plot, two typical vegetation communities (*P. hydropiper* community and *T. lutarioriparia* community) were selected at two heights (high and low) above the water table. The different heights (high and low) above the water table were defined according to the elevation. Thus, soil samples collected from the two typical vegetation communities at two heights above the water table could be divided into four experiment groups: *P. hydropiper* community at a high level above the water table (HP), *T. lutarioriparia* community at a high level above the water table (HT), *P. hydropiper* community at a low level above the water table (LP), and the *T. lutarioriparia* community at a low level above the water table (LT) (Figure 1). At the same level above the water table in each sample plot, three soil samples collected from the *P. hydropiper* community were mixed as a soil sample, and three soil samples collected from the *T. lutarioriparia* community were mixed as a soil sample. A total of 12 mixed soil samples were collected. The surface sundries were removed when collecting the samples. The topsoil (0–15 cm) was collected as the soil sample owing to its high organic carbon content and packed in self-sealing bags for low-temperature preservation. In the laboratory, each soil sample was divided into three parts, one part was stored in the refrigerator at −20 °C to measure microbial diversity, one part was stored in the refrigerator at 4 °C to measure biomass carbon and nitrogen, and one part was air-dried to measure soil physical-chemical properties and organic carbon characteristics. The samples used for DNA extraction were stored at −20 °C for about 2 days, and samples used for biomass carbon and nitrogen analysis were stored at 4 °C for about 3 days.

### 2.3. Determination of Soil Physicochemical Properties and Organic Carbon Characteristics

The air-dried soil samples were passed through a 2 mm sieve to remove plant residues and stones, and roots were carefully picked out. Then, the samples were divided into three parts: one part was used for the measurements of soil pH and soil water content (WC) and the other was passed through a 0.15 mm sieve for soil total nitrogen (TN), total carbon (TC), total phosphorus (TP), organic carbon (SOC), and particulate organic carbon (POC) contents analyses. For recombinant organic carbon (HFOC) and light group organic carbon (LFOC) contents analyses, the soil samples were passed through a 0.25-mm sieve. The soil pH was determined by potentiometric method (water: soil = 1:5; *w*/*v*) with a pH meter (PHS-3D, Shanghai Leica Instrument Ltd. Co., Shanghai, China), and the soil WC was ascertained by drying method using an oven at 105 °C for 10 h. The TN and TC contents were measured by an element analyzer (Vario MACRO cube, Elementar Trading Shanghai, Shanghai, China), while the TP content was evaluated by NaOH alkali melting molybdenum antimony anti colorimetry using a spectrophotometer (AA900T, Perkin Elmer, Norwalk, CA, USA) [24]. The SOC content was determined by potassium dichromate colorimetry with a spectrophotometer (AA900T, Perkin Elmer, Norwalk, CA, USA) [25]. The POC content was evaluated according to the method developed by Sun et al. [26]. HFOC and LFOC contents were ascertained by using the density method [27]. The fresh soil stored at 4 °C was passed through a 2 mm sieve for the measurement of soluble organic carbon (DOC), microbial biomass carbon (MBC), and microbial biomass nitrogen (MBN) contents. The MBC and MBN contents were measured by the chloroform fumigation method [28]. DOC content was determined using a TOC analyzer (TOC-L, Shimadzu Scientific Instruments, Columbia, ML, USA). 

### 2.4. Soil DNA Extraction and PCR Amplification

Soil DNA was extracted from 0.50 g of fresh soil by using a metagenomic DNA Extraction Kit (GENErary) according to the manufacturer’s instructions. The quality of the extracted DNA was detected by 1% agarose gel electrophoresis, and the DNA integrity was ascertained. Subsequently, the purity and concentration of the extracted DNA were determined by UV spectrophotometer, and the DNA was frozen in a refrigerator at −20 °C. The V3-V4 hypervariable region of the bacterial 16S rRNA gene and fungal ITS1 gene were amplified by real-time fluorescence quantitative method on a PCR instrument (EDC-810; Beijing Dongsheng Innovation Biotechnology Co., Ltd., Beijing, China). The bacterial amplification primers were 338f (ACTCCTACGGGAGGCAGCAG) and 806r (GGACTACHVGGGTWTCTAAT) [29]. The fungal amplification primers were ITS1F (CTTGGTCATTTAGAGGAAGTAA) and ITS2 (GCTGCGTTCTTCATCGATGC) [30]. The reaction system (16 μL) included 7 μL of SYBR Green Mix, 0.5 μL of Primer-F, 0.5 μL of Primer-R, and 8 μL of cDNA. The amplification procedure was as follows: pre-denaturation at 95 °C for 10 min, 40 cycles of denaturation at 95 °C for 10 s, annealing at 60 °C for 34 s, extension at 72 °C for 30 s, and a final extension at 72 °C for 10 min and cooled to 10 °C for amplification.

### 2.5. High-throughput Sequencing of Bacterial 16S rRNA Gene and Fungal ITS1 Gene

The amplicons of the amplified products were extracted by using 2% agarose gel, purified by a Gel Recovery Kit, and sequenced by the ILPuminaMiSeq platform (Beijing Bai Mai Biotechnology, Co. Ltd., Beijing, China). The original data were filtered by trimmatic tool, and the primer sequences were identified and removed by Cutadapt software according to the parameters of 20% maximum mismatch rate and 80% minimum coverage. FLASH v1.2.11 software was employed to splice the reads of each sample according to the minimum overlap length of 10 bp, and Lima v1.7.0 software was used to identify the circular consensus sequences (CCS) of different samples through barcode sequence and remove chimeras to obtain high-quality CCS. Subsequently, USEARCH was applied to cluster the sequences at 97% similarity level and filter the operational taxonomic units (OTUs) with 0.005% as the threshold for the number of sequences. The characteristic sequence was compared with the Greengenes database with the classify consumption blast in QIIME2 (http://greengenes.secondgenome.com/, accessed on 30 August 2021). If the characteristic sequences could not be accurately compared with the reference database, then the Classfy-Sklearn classifier was used. The threshold according to the similarity between the sequences was divided into OTUs for species annotation. The relative abundance of bacteria and fungi in the samples was evaluated at the phylum, class, order, family, genus, and species levels. Based on the OTUs, species richness (ACE and Chao1 index), diversity (Simpson and Shannon index), and evenness (Pielou index) were calculated using alpha index analysis software QIIME2 (https://qiime2.org/, accessed on 3 September 2021).

### 2.6. Data Analysis

The data obtained were analyzed using SPSS 19.0 (SPSS Inc., Chicago, IL, USA). The Duncan method was used to perform multiple comparisons of means (*p* < 0.05). Pearson’s correlation analysis was performed to ascertain the correlation of soil physicochemical properties, SOC characteristics, and soil microbial diversity-related indices. Two-way ANOVA was conducted to determine the effects of vegetation type and water level on indices. The results are expressed as the mean ± standard error. The number of soil bacterial and fungal OTUs was transformed by log normalization. The R 3.6.2 software was used to construct a relative abundance map of dominant species at the phylum and class levels. Canoco 5.0 was employed to derive the canonical correspondence analysis (CCA) map of the bacterial and fungal communities and environmental indices (soil physicochemical properties and SOC characteristics).

## 3. Results and Analysis

### 3.1. Soil Physicochemical Properties and Organic Carbon Characteristics

Two-way ANOVA showed that the impact of water level on SOC characteristics was higher than that of vegetation type. Vegetation type had a significant effect only on soil DOC, whereas water level had a significant impact on soil WC, TN, C/N, SOC, HFOC, DOC, and MBC (Table 1). There was no significant difference in soil pH, TP, TC, LFOC, POC, and MBN among different groups. The levels of TN, SOC, HFOC, and MBC among the different groups presented a consistent trend as follows: LP > LT > HT > HP, and the values were higher in soil at a low level above the water table than those at a high level above the water table. However, C/N was higher in soil at a high level above the water table, and the value was significantly lower in LP soil than that in other groups. The DOC content in LP soil was significantly higher than that in other groups.

### 3.2. Microbial α-Diversity

A total of 127,442 bacterial gene sequences were detected in the soils of all four groups, and 1288 bacterial OTUs were obtained. Two-way ANOVA showed that the soil bacterial OTUs and richness (ACE and Chao1) were predominantly affected by vegetation type, while the soil bacterial diversity (Simpson and Shannon) was mainly affected by the water level (Table 2). In contrast, soil bacterial sequence and evenness (Pielou) were affected by both vegetation type and water level. The bacterial OTUs and richness in *T. lutarioriparia* community soils were higher than those in *P. hydropiper* community soils, and the difference was significant at a high level above the water table. The bacterial sequence in soil at a low level above the water table was significantly higher than that at a high level above the water table. However, the bacterial diversity and evenness were significantly higher in soil at a high level above the water table than those at a low level above the water table and showed the following trend: HP > HT > LT > LP. In addition, bacterial evenness in *P. hydropiper* community soils was higher than that in *T. lutarioriparia* community soils.

A total of 320,882 fungal gene sequences were detected in the soils of the four groups, and a total of 634 fungal OTUs were obtained. Two-way ANOVA showed that vegetation type and water level had significant effects on soil fungal OTUs, sequence, richness (ACE and Chao1), diversity (Simpson and Shannon), and evenness (Pielou) (Table 3). The soil fungal OTUs at a low level above the water table were higher than those at a high level above the water table, and the difference was significant in *P. hydropiper* community soils. The fungal sequence in *P. hydropiper* community soil at a high level above the water table was significantly higher than that at a low level above the water table. The effect of vegetation type on soil fungal richness was higher than that of water level, and the fungal richness in *T. lutarioriparia* community soils was higher than that in *P. hydropiper* community soils. Furthermore, the fungal richness in *P. hydropiper* community soil at a low level above the water table was significantly higher than that at a high level above the water table. The fungal diversity and evenness in different soil groups presented the following trend: LT > LP > HT > HP, and were significantly higher at a low level above the water table than those at a high level above the water table. In addition, the fungal diversity and evenness were significantly higher in *P. hydropiper* community soil than those in *T. lutarioriparia* community soil under the same water level.

### 3.3. Microbial ß-Diversity

**Figure 2:**
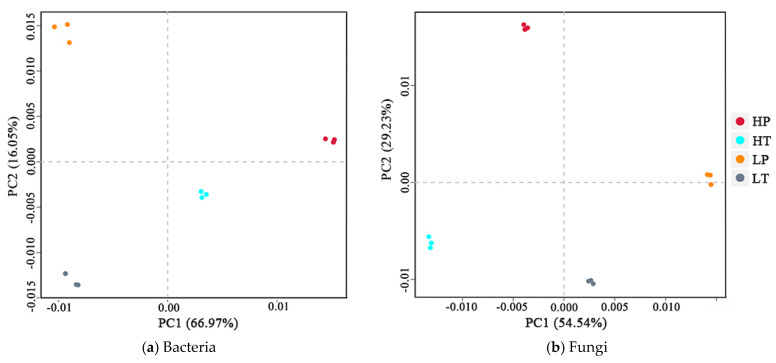
Principal component analysis (PCA) of soil microbial communities in different groups. HP denotes *P. hydropiper* community at high level above the water table, HT signifies *T. lutarioriparia* community at high level above the water table, LP indicates *P. hydropiper* community at low level above the water table, and LT refers to *T. lutarioriparia* community at low level above the water table.

The differences in microbial OTUs composition in soil samples from the four groups can be reflected in two-dimensional coordinates using principal component analysis (PCA), and the differences and distances of OTUs in various positions can be obtained. As shown in Figure 2a, the first axis (PCA1) and second axis (PCA2) of the principal component could explain 66.97% and 16.05% of the variation in the soil bacterial community, respectively. HP occurred in the positive direction of PCA1 and PCA2, HT occurred in the positive direction of PCA1 and negative direction of PCA2, LP occurred in the negative direction of PCA1 and positive direction of PCA2, and LT occurred in the negative direction of PCA1 and PCA2. As shown in Figure 2b, PCA1 and PCA2 could explain 54.54% and 29.23% of the variation in the fungal community, respectively, with HP in the negative direction of PCA1 and positive direction of PCA2, HT in the negative direction of PCA1 and PCA2, LP in the positive direction of PCA1 and PCA2, and LT in the positive direction of PCA1 and negative direction of PCA2. In general, with regard to soil bacterial community, samples collected at a high level above the water table remained in the positive direction of PCA1, while those collected at a low level above the water table were in the negative direction of PCA1. Furthermore, *P. hydropiper* community soil samples occurred in the positive direction of PCA2, whereas *T. lutarioriparia* community soil samples remained in the negative direction of PCA2. With respect to the soil fungal community, samples collected at a high level above the water table occurred in the negative direction of PCA1, while those collected at a low level above the water table were in the positive direction of PCA1. Moreover, *P. hydropiper* community soil samples occurred in the positive direction of PCA2, whereas *T. lutarioriparia* community soil samples were in the negative direction of PCA2. These results indicated that both water level and vegetation type had a significant effect on soil bacterial and fungal communities, with water level exerting a higher effect on soil bacterial and fungal communities than vegetation type. The changes in the soil bacterial and fungal community showed the opposite trend at different water levels but were consistent under different vegetation types. 

### 3.4. Microbial Community Composition

**Figure 3:**
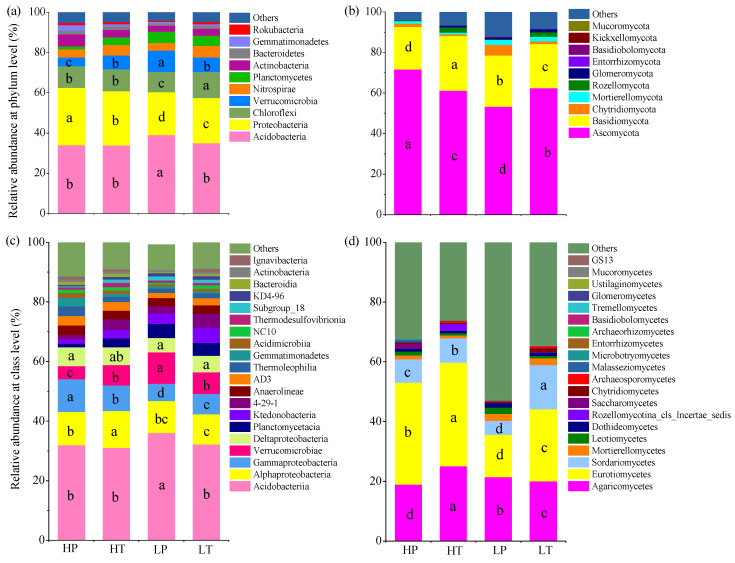
Relative abundances of soil microbial communities at phylum and class levels in different groups. (**a**) Bacteria at phylum level; (**b**) fungi at phylum level; (**c**) bacteria at class level; (**d**) fungi at class level. HP denotes *P. hydropiper* community at high level above the water table, HT indicates *T. lutarioriparia* community at high level above the water table, LP signifies *P. hydropiper* community at low level above the water table, and LT refers to *T. lutarioriparia* community at low level above the water table. Different lowercase letters represent significant differences among groups (*p* < 0.05).

A total of 29 phyla, 70 classes, 149 orders, 221 families, 293 genera, and 298 species of bacteria were identified in the soil samples of the four groups. The relative abundance of the top 10 bacterial phyla, including Acidobacteria, Proteobacteria, Chloroflexi, Verrucomicrobia, Nitrospirae, Actinobacteria, Planctomycetes, Bacteroidetes, Gemmatimonadetes, and Rokubacteria, was 95.23% in total (Figure 3a). Among them, the dominant bacterial phyla were Acidobacteria (35.47%), Proteobacteria (24.80%), Chloroflexi (11.16%), and Verrucomicrobia (7.22%), which presented a relative abundance of 78.66% in total. The dominant bacterial classes were Acidobacteriia (32.84%), Alphaproteobacteria (11.08%), Gammaproteobacteria (8.07%), Verrucomicrobia (7.22%), and Deltaproteobacteria (5.65%), which accounted for 64.86% of relative abundance in total (Figure 3c). The relative abundances of Acidobacteria and Acidobacteriia were higher at a low level above the water table than those at a high level above the water table and were significantly higher in LP soil. Similarly, the relative abundances of Verrucomicrobia were higher at a low level above the water table than those at a high level above the water table. In contrast, the relative abundances of Proteobacteria (*p* < 0.05), Alphaproteobacteria, and Gammaproteobacteria (*p* < 0.05) were higher at a high level above the water table than those at a low level above the water table. Moreover, the relative abundance of Chloroflexi in *T. lutarioriparia* community soils was higher than that in *P. hydropiper* community soils. 

A total of 11 phyla, 24 classes, 52 orders, 82 families, 98 genera, and 80 species of fungi were identified in the soil samples of the four groups. The relative abundance of the top five fungal phyla, including Ascomycota, Basidiomycota, Chytridiomycota, Mortierellomycota, and Rozellomycota, accounted for 91.15% in total (Figure 3b). Among them, Ascomycota (62.11%) and Basidiomycota (23.77%) were the dominant fungal phyla, accounting for 85.88% of the total relative abundance. Eurotiomycetes (26.78%), Agaricomycetes (21.36%), and Sordariomycetes (8.52%) were the dominant fungal classes, accounting for 57.00% of the total relative abundance (Figure 3d). The relative abundance of Ascomycota in different groups presented the following trend: HP > LT > HT > LP, while that of Basidiomycota and Agaricomycocetes showed the following trend: HT > LP > LT > HP. The relative abundance of Eurotiomycetes was higher at a high level above the water table than that at a low level above the water table, and was higher in *T. lutarioriparia* community soil than that in *P. hydropiper* community soil under the same water level. Furthermore, the relative abundance of Sordariomycetes in *T. lutarioriparia* community soil was significantly higher than that in *P. hydropiper* community soil.

### 3.5. Microbial Diversity and Environmental Indices

Correlation analysis showed soil bacterial richness (Chao1) had a significant relationship only with TP (r = 0.590, *p* < 0.05), whereas fungal richness (ACE and Chao1) had a significant positive correlation with WC, LFOC, MBC, and MBN, with the highest correlation noted with WC (r = 0.745, *p* < 0.01; r = 0.768, *p* < 0.01) (Table 4). Soil bacterial diversity (Simpson and Shannon) and evenness (Pielou) were significantly positively correlated with C/N (*p* < 0.01), and negatively correlated with WC (*p* < 0.01), TN (*p* < 0.01), HFOC (*p* < 0.01), SOC (*p* < 0.01), DOC (*p* < 0.01), TC (*p* < 0.01), MBC (*p* < 0.05), and POC (*p* < 0.05). In addition, soil bacterial evenness was significantly negatively correlated with LFOC and MBN (*p* < 0.05), whereas soil fungal diversity and evenness were positively correlated with WC (*p* < 0.01), MBC (*p* < 0.01), POC (*p* < 0.05), TN (*p* < 0.05), TC (*p* < 0.05), HFOC (*p* < 0.05), and SOC (*p* < 0.05). Furthermore, the soil fungal evenness (Pielou) was also positively correlated with LFOC and MBN (*p* < 0.05).

### 3.6. Microbial Community and Environmental Indices

**Figure 4:**
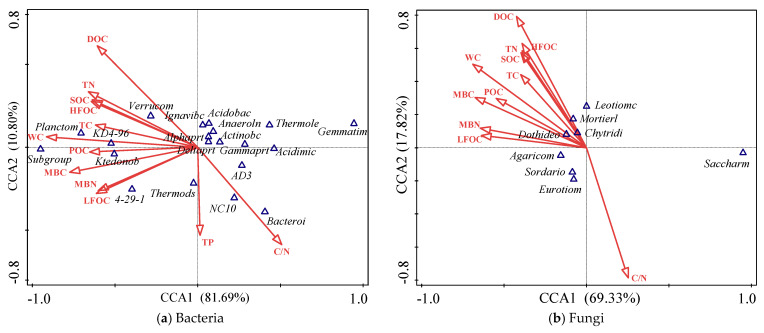
Canonical correspondence analysis (CCA) of soil microbial communities and environmental indices. *Acidimic:* Acidimicrobiia, *Acidobac*: Acidobacteriia, *Actinobc*: Actinobacteria, *Alphaprt*: Alphaproteobacteria, *Anaeroln*: Anaerolineae, *Bacteroi*: Bacteroidia, *Deltaprt*: Deltaproteobacteria, *Gammaprt*: Gammaproteobacteria, *Gemmatim*: Gemmatimonadetes, *Ignavibc*: Ignavibacteria, *Ktedonob*: Ktedonobacteria, *Planctom*: Planctomycetacia, *Subgroup*: Subgroup_18, *Thermods*: Thermodesulfovibrionia, *Thermole*: Thermoleophilia, *Verrucom*: Verrucomicrobiae; *Agaricom*: Agaricomycetes, *Chytridi*: Chytridiomycetes, *Dothideo*: Dothideomycetes, *Eurotiom*: Eurotiomycetes, *Leotiomc*: Leotiomycetes, *Mortierl*: Mortierellomycetes, *Sordario*: Sordariomycetes, *Saccharm*: Saccharomycetes.

CCA can be used to study the relationship between environmental indices (soil physicochemical properties and SOC characteristics) and microbial community. In this study, the first axis (CCA1) and second axis (CCA2) of the ranking axis could explain 81.69% and 10.80% of the variation in the bacterial community, respectively (Figure 4a), and 69.33% and 17.82% of the variation in the fungal community, respectively (Figure 4b). The main environmental indices affecting the soil bacterial community were soil WC, DOC, C/N, MBC, etc. (Figure 4a). Acidobacteria was positively associated with soil WC, TN, DOC, SOC, HFOC, TC, MBC, and TC, but negatively correlated with soil C/N. Alphaproteobacteria was positively correlated with soil LFOC, MBC, and WC. Gammaproteobacteria was negatively related to soil WC, DOC, TN, C/N, HFOC, SOC, MBC, POC, and TC. Verrucomicrobia was positively associated with soil WC, DOC, TN, SOC, HFOC, MBC, TC, and DOC, but extremely significantly negatively correlated with soil C/N. The most important environmental factors affecting soil fungal community were soil DOC, C/N, WC, MBC, etc. (Figure 4b). Eurotiomycetes were extremely significantly positively correlated with soil C/N but negatively associated with soil DOC, WC, TN, and SOC. There was no significant correlation between the relative abundances of Agaricomycetes and Sordariomycetes with the soil factors.

## 4. Discussion

### 4.1. Effects of Water Level and Vegetation Type on SOC Characteristics in Wetland

In general, SOC can be divided into activated carbon pool, slow carbon pool, and inert carbon pool according to the turnover time. Among them, soil active organic carbon, including DOC, MBC, mineralizable carbon (EOC), LFOC, POC, etc., is the part of organic carbon that moves fast, has poor stability, and is easy to oxidize and mineralize in the soil [31]. Although the proportion of soil active organic carbon in SOC is small, it is more sensitive to the environment and can better reflect the changes in the soil carbon pool, and thus has a significant impact on soil carbon dynamics and can indicate global climate change [32]. The SOC characteristics in wetlands are affected by many factors, such as wetland type, hydrological conditions, vegetation type, land use type, soil nutrients, soil pH, soil salt content, etc. Water level is the key factor that affects SOC in wetlands. The increase in the groundwater level can reduce the oxygen content in soil and decrease microbial activity, inhibit microbial and plant root respiration, and ultimately reduce microbial decomposition of organic carbon, which is conducive to soil carbon accumulation [33,34]. Similarly, the results of the present study also indicated that water level had significant effects on soil WC, TN, C/N, SOC, HFOC, DOC, and MBC. The contents of SOC, HFOC, and MBC in soils at a low level above the water table were higher than those in soils at a high level above the water table, presenting a significant positive correlation with soil WC. The effect of low water levels has also been reported, which was related to flooding time, and long-term flooding has been found to be more conducive to the accumulation of organic matter [11]. The decrease in the water level in the Poyang Lake wetland owing to climate change and human activities has caused a significant reduction in wetland area as well as advancement and extension of exposure time of wetland beach, ultimately reducing the soil carbon sequestration capacity. Vegetation type is also an important factor that affects carbon storage and carbon fixation in wetland ecosystems. Plant litter is the main carbon source in wetlands, and most of the active organic components of wetland soil originate from plants [35]. Wang et al. found that vegetation significantly affected the variation in SOC and DOC in the Poyang Lake wetland [36]. The results of the present study showed that vegetation type had no significant effect on soil SOC, HFOC, LFOC, POC, and MBC, but significantly affected soil DOC. This might be attributed to the fact that DOC mainly originates from plant litter and root exudates, and the composition and quantity of litter and root exudates are different between *T. lutarioriparia* and *P. hydropiper*.

### 4.2. Effects of Water Level and Vegetation Type on Soil Bacterial and Fungal Communities in Wetland

High-throughput sequencing technology is a new-generation sequencing technique, which can comprehensively and accurately determine the microbial community characteristics. However, studies using this technology on soil microorganisms in wetland ecosystems are limited. When compared with other ecosystems, the wetland ecosystem comprises unique microbial communities owing to its high soil WC and distinctive vegetation types. Studies on soil microbial communities based on high-throughput sequencing have revealed that the relative abundance of dominant microorganisms among different types of wetlands can significantly vary. For instance, the dominant bacteria in the soil of Zoige Plateau were reported to be Proteobacteria (36.50%), Acidobacteria (26.10%), Actinobacteria (9.40%), and Bacteroidetes (5.80%) [37]. In the soil of the coastal mangrove salt marsh ecosystem, Proteobacteria (55.00%), Bacteroidetes (9.80%), Acidobacteria (4.00%), Chloroflexi (3.40%), and Nitrospirae (3.00%) were found to be the dominant bacteria, and Ascomycota (37.00%) and Basidiomycota (5.60%) were noted to be the dominant fungi [27]. The present study revealed that the dominant bacteria in the Poyang Lake wetland soil were Acidobacteria (35.47%), Proteobacteria (24.80%), Chloroflexi (11.16%), and Verrucomicrobia (7.22%), while the dominant fungi were Ascomycota (62.11%) and Basidiomycota (23.77%). As different types of wetlands exhibit obvious differences in hydrology, vegetation, climate, and soil, the relative abundance of dominant soil microorganisms can vary. In the present study, the most dominant bacteria in Poyang Lake wetland soil were found to be Acidobacteria, rather than Proteobacteria, which may be owing to the WC of the soil samples. It must be noted that the WC of the samples in this study was less than 30%, whereas that of the soil samples of the Zoige Plateau and coastal mangrove salt marsh ecosystem was reported to be more than 90%. As a result, the soil oxygen content in the present study was higher, and hence, the abundance of Proteobacteria, which are mostly facultative or obligate anaerobic and heteroaerobic, was not high in the present study.

Furthermore, the richness and evenness of bacteria in the Poyang Lake wetland were found to be much higher than those of fungi because bacteria have more survival advantages under the humid condition of high groundwater level gradient [38]. On a regional scale, water level and vegetation type are the main factors that affect the wetland microbial community [39]. The results of the present study showed that vegetation types had a significant impact on the richness index of bacteria and fungi. The richness of bacteria and fungi in *T. lutarioriparia* community soils was higher than that in *P. hydropiper* community soils. This may be owing to the higher types or contents of root exudates of *T. lutarioriparia* than those of *P. hydropiper*, which can provide more varieties of carbon sources for microbial growth, thus causing an increase in microbial richness. In addition, the water level significantly affected the diversity of bacteria and fungi, mainly because the increase in water level can lead to the increase in soil WC and decrease in soil oxygen content, which are conducive to anaerobes but not to aerobes. At the same time, the increase in groundwater level can also alter the soil nutrients and affect the microbial community [40], which was confirmed in the present study by the results of correlation analysis that indicated that the diversity and richness of soil bacterial and fungal communities in the Poyang Lake wetland were significantly correlated with soil WC. Furthermore, water level and vegetation type also significantly affected the structure of soil microbial community. PCA showed that the impact of water level on soil bacterial and fungal community was higher than that of vegetation type, and the changes in the bacterial and fungal communities were opposite at different water levels. Water level predominantly affects the microbial community owing to the changes in soil WC. Fan et al. found that soil moisture was the main factor affecting the soil microbial community and diversity in the Zoige Plateau [37], while Li et al. reported that soil moisture and salt content were the main factors affecting soil microbial community in the humid land of Chongming east beach [41]. Furthermore, Zhang et al. investigated the soil microbial community in the Poyang Lake wetland by the phospholipid fatty acid method and noted that soil pH, WC, and mechanical composition were the main factors affecting the structure of the soil microbial community [21]. In the present study, correlation analysis and CCA revealed that water level was the main soil factor affecting the changes in the soil microbial community in Poyang Lake wetland. Moreover, the soil bacterial diversity index at high level above the water table in Poyang Lake wetland was significantly higher than that at low level above the water table, whereas the soil fungal diversity showed the opposite trend, which was consistent with the findings reported by Cook et al. [42]. The reason for this observation could be owed to the fact that bacteria are more active at high levels above the water table, whereas fungi are more active at low levels above the water table. The diversity and richness of soil bacterial community were significantly negatively correlated with soil WC, whereas those of soil fungal community were positively correlated with soil WC, which was mainly owing to the predominant effects of soil WC and SOC on wetland soil microorganisms [43]. It must be noted that bacterial respiration is easily inhibited by the increase in soil moisture content, whereas fungi are more susceptible to the availability of carbon sources.

Among bacteria with high relative abundance, Gammaproteobacteria was significantly negatively correlated with soil WC, whereas Alphaproteobacteria and Verrucomicrobia were extremely significantly positively correlated with soil WC. Besides, Gemmatimonadetes, Thermoleophilia, Acidimicrobiia, AD3, Planctomycetacia, Subgroup_18, Acidobacteriia, KD4-96, and Ktedonobacteria were significantly correlated with WC and were the key bacterial groups that indicated the changes in wetland water level. The key fungal groups signifying the changes in water level in the wetland were Saccharomycetes, Eurotiomycetes, and Dothideomycetes, which were significantly correlated with soil WC. The relative abundances of Chloroflexi, Eurotiomycetes, and Sordariomycetes were significantly higher in *T. lutarioriparia* community soil than that in *P. hydropiper* community soil, suggesting that they were the key microbiota indicating the vegetation types (*T. lutarioriparia* and *P. hydropiper*).

### 4.3. Relationship between SOC Characteristics and Microbial Community in Wetland

Although organic carbon is another limiting factor that affects wetland soil microbial community [43,44], there are only a few studies on the relationship between wetland SOC characteristics and soil microorganisms, which limits the understanding of the microbial-driven mechanism of the wetland soil carbon cycle. The results of the present study showed that the SOC and TN contents were significantly negatively correlated with soil bacterial richness, diversity, and evenness indices. At the same time, the SOC was significantly positively correlated with soil moisture content, because the soil oxygen content decreases with the increasing soil WC, resulting in the reduction in aerobic microorganisms and a decline in microbial activity, ultimately decreasing the decomposition and promoting the accumulation of organic carbon. Soil C/N can reflect whether microbial growth is carbon-limited or nitrogen-limited, and carbon availability decreases with increasing the C/N ratio. In the present study, soil C/N was significantly positively correlated with wetland soil bacterial diversity, and significantly negatively correlated with soil WC, which further demonstrated that soil bacterial diversity in wetlands is limited by carbon availability, and carbon source is affected by soil WC. With regard to soil fungi, all the organic carbon characteristics, except DOC, were significantly positively correlated with fungal diversity. When the soil WC is relatively low, the number of soil fungi increases with increasing SOC content. In contrast, when the soil WC is high or flooded, the increase in soil WC inhibits the growth and reproduction of fungi [45]. In the present study, when the soil WC was low (<30%), the organic carbon characteristics and available carbon source for fungi increased with the increasing soil WC, and hence, the fungal diversity increased. Furthermore, organic carbon characteristics could affect the wetland microbial community. Among the dominant microbial communities, Acidobacteriia, Alphaproteobacteria, and Verrucomicrobia were significantly positively correlated with organic carbon characteristics, whereas Gammaproteobacteria and Eurotiomycetes were significantly negatively correlated with organic carbon characteristics, and these microorganisms are the key microbiota of the soil carbon cycle in the Poyang Lake wetland.

## 5. Conclusion

Although soil SOC and microbial community in the Poyang Lake wetland were affected by both water level and vegetation type, the impact of water level was much higher than that of vegetation type. The diversity and richness of bacteria in wetland soil were much higher than those of fungi, and the sensitivity of fungal diversity to water level was higher than that of bacterial diversity. The regulation of wetland carbon fixation by water level is accomplished through improving the survival pressure of bacteria mainly by affecting soil WC, which in turn influences the bacterial community structure and diversity, ultimately causing changes in the organic carbon characteristics. In the range of the soil WC examined in this study, the water level was found to be the limiting factor of wetland soil bacteria, whereas organic carbon characteristics were noted to be the limiting factor of wetland soil fungi. The findings of this study could help in achieving a further understanding of the role of microorganisms in soil carbon storage in the Poyang Lake wetland.

## Figures and Tables

**Table 1:** Soil physicochemical properties and organic carbon characteristics in different groups.

Group	pH	WC (%)	TP (g kg^−1^)	TC (%)	TN (g kg^−1^)	C/N	SOC (g kg^−1^)	HFOC (g kg^−1^)	LFOC (g kg^−1^)	POC (g kg^−1^)	DOC (g kg^−1^)	MBC (g kg^−1^)	MBN (g kg^−1^)
HP	5.05a	20.59b	0.417a	0.681a	0.503b	13.55a	5.27b	3.776b	0.832a	1.544a	0.109b	0.926b	0.018a
HT	5.15a	25.53a	0.454a	0.928a	0.701ab	13.35a	7.30ab	5.38ab	1.392a	3.655a	0.169b	1.420a	0.030a
LP	5.02a	28.15a	0.394a	1.190a	1.111a	10.78b	11.03a	8.138a	1.289a	4.259a	0.370a	1.497a	0.029a
LT	4.97a	26.81a	0.446a	1.076a	0.835ab	13.08a	8.55ab	6.35ab	1.322a	3.922a	0.150b	1.453a	0.028a
Significance based on Two-way ANOVA (*p*)													
VT	0.784	0.077	0.266	0.697	0.787	0.085	0.882	0.935	0.104	0.327	**0.034**	0.110	0.155
WL	0.230	**0** **.001**	0.682	0.080	**0.029**	**0** **.021**	**0.045**	**0.044**	0.266	0.118	**0.005**	**0.042**	0.260
VT × WT	0.378	**0** **.008**	0.852	0.304	0.128	0.047	0.164	0.167	0.141	0.188	**0.002**	0.064	0.118

Note: HP denotes *P. hydropiper* community at high level above the water table, HT indicates *T. lutarioriparia* community at high level above the water table, LP signifies *P. hydropiper* community at low level above the water table, LT indicates *T. lutarioriparia* community at low level above the water table, VT refers to vegetation type, and WL refers to water level. Different lowercase letters represent significant differences among groups (*p* < 0.05).

**Table 2:** The α-diversity index of bacteria in different groups.

Group	OTU_Num	Seqs_Num	Richness		Diversity		Evenness
			ACE	Chao1	Simpson	Shannon	Pielou
HP	1003 ± 7b	9273 ± 9.2d	1137 ± 19c	1143 ± 23.2b	0.995 ± 0.000a	8.714 ± 0.008a	1.261 ± 0.001a
HT	1080 ± 13a	10,116 ± 40c	1238 ± 16a	1223 ± 12a	0.994 ± 0.000b	8.612 ± 0.029b	1.233 ± 0.002b
LP	1010 ± 10b	11,307 ± 11b	1149 ± 12bc	1145 ± 9b	0.992 ± 0.000d	8.298 ± 0.020d	1.200 ± 0.001d
LT	1063 ± 3a	11,785 ± 17a	1191 ± 13ab	1185 ± 17ab	0.993 ± 0.000c	8.485 ± 0.021c	1.218 ± 0.003c
Significance based on Two-way ANOVA							
VT	**0.000**	**0.000**	**0.002**	**0.006**	0.587	0.074	**0.049**
WL	0.577	**0.000**	0.281	0.295	**0.000**	**0.000**	**0.000**
VT × WT	0.198	**0.000**	0.084	0.258	**0.000**	**0.000**	**0.000**

Note: HP signifies *P. hydropiper* community at high level above the water table, HT denotes *T. lutarioriparia* community at high level above the water table, LP indicates *P. hydropiper* community at low level above the water table, LT refers to *T. lutarioriparia* community at low level above the water table, VT indicates vegetation type, and WL refers to water level. Different lowercase letters represent significant differences among groups (*p* < 0.05).

**Table 3:** The α-diversity index of fungi in different groups.

Group	OTU_Num	Seqs_Num	Richness		Diversity		Evenness
			ACE	Chao1	Simpson	Shannon	Pielou
HP	377 ± 2c	27,069 ± 18a	413 ± 4b	419 ± 5b	0.907 ± 0.001d	4.813 ± 0.008d	0.811 ± 0.001d
HT	427 ± 3b	26,771 ± 25b	472 ± 5a	475 ± 5a	0.927 ± 0.001c	5.284 ± 0.015c	0.873 ± 0.002c
LP	439 ± 3a	26,321 ± 35c	456 ± 4ab	464 ± 6a	0.943 ± 6.667b	5.47 ± 0.005b	0.900 ± 0.002b
LT	430 ± 2b	26,799 ± 29b	461 ± 4ab	469 ± 39a	0.947 ± 0.000a	5.574 ± 0.005a	0.919 ± 0.002a
Significance based on Two-way ANOVA							
VT	**0.000**	**0.011**	**0.000**	**0.000**	**0.000**	**0.000**	**0.000**
WL	**0.000**	**0.000**	**0.006**	**0.003**	**0.000**	**0.000**	**0.000**
VT × WT	**0.000**	**0.000**	**0.000**	**0.001**	**0.000**	**0.000**	**0.000**

Note: HP denotes *P. hydropiper* community at high level above the water table, HT indicates *T. lutarioriparia* community at high level above the water table, LP signifies *P. hydropiper* community at low level above the water table, LT denotes *T. lutarioriparia* community at low level above the water table, VT refers to vegetation type, and WL indicates water level. Different lowercase letters represent significant differences among groups (*p* < 0.05).

**Table 4:** Pearson’s correlation of microbial α-diversity index and environmental indices.

Index	Bacteria					Fungi				
	ACE	Chao1	Simpson	Shannon	Pielou	ACE	Chao1	Simpson	Shannon	Pielou
pH	0.232	0.154	0.363	0.319	0.262	0.090	0.053	−0.240	−0.194	−0.224
TN	−0.007	−0.015	−0.822 **	−0.831 **	−0.805 **	0.410	0.468	0.624 *	0.611 *	0.598 *
TP	0.551	0.590 *	0.179	0.247	0.106	−0.003	−0.012	0.004	0.060	0.084
TC	0.167	0.166	−0.717 **	−0.693 *	−0.714 **	0.419	0.473	0.597 *	0.604 *	0.603 *
WC	0.269	0.221	−0.912 **	−0.852 **	−0.926 **	0.745 **	0.768 **	0.863 **	0.879 **	0.863 **
C/N	0.320	0.333	0.742 **	0.814 **	0.708 **	−0.229	−0.258	−0.462	−0.414	−0.384
SOC	0.040	0.037	−0.783 **	−0.787 **	−0.772 **	0.379	0.432	0.599 *	0.592 *	0.584 *
HFOC	0.039	0.038	−0.790 **	−0.790 **	−0.777 **	0.397	0.454	0.607 *	0.601 *	0.592 *
LFOC	0.465	0.410	−0.521	−0.474	−0.585 *	0.657 *	0.718 **	0.558	0.613 *	0.600 *
POC	0.317	0.328	−0.693 *	−0.622 *	−0.694 *	0.559	0.554	0.610 *	0.638 *	0.631 *
DOC	−0.195	−0.229	−0.758 **	−0.815 **	−0.750 **	0.308	0.294	0.494	0.466	0.433
MBC	0.513	0.488	−0.707 *	−0.638 *	−0.759 **	0.687 *	0.694 *	0.725 **	0.768 **	0.756 **
MBN	0.513	0.494	−0.527	−0.462	−0.577 *	0.605 *	0.585 *	0.527	0.590*	0.577 *

Note: ** *p* < 0.01; * *p* < 0.05.

## Data Availability

Data could be obtained upon request to the authors.
